# Low Nest Success of Black‐Legged Kittiwakes (*Rissa tridactyla*) at a Mainland Colony Is Not Associated With Disturbance From Walkers or Boats: Implications for Compensation for Offshore Renewables Impacts

**DOI:** 10.1002/ece3.74337

**Published:** 2026-09-13

**Authors:** Patrick J. C. White, Hannah Humphreys, Jill M. Grozier, Jenna R. Lane, Alexander M. Willey, Annalise Bokenkamp, Nils H. Schoefer, Sophie J. Wilson, Ross J. Rutherford, Liza Cole, Ciaran Hatsell, Rachel Bonnici, Ellie Owen, Michelle Frost, Sue Lewis, Karen Diele

**Affiliations:** ^1^ Centre for Conservation & Restoration Science Edinburgh Napier University Edinburgh UK; ^2^ School of Applied Sciences Edinburgh Napier University Edinburgh UK; ^3^ National Trust for Scotland St Abbs UK

**Keywords:** breeding success, compensation, demography, disturbance, mitigation, offshore windfarms, protected area, seabirds

## Abstract

Seabirds face a range of pressures including rising sea temperatures, declining food availability, offshore renewables and disturbances at their onshore nesting colonies. Developers of marine renewables often seek to implement compensatory measures to offset risk and enhance productivity through targeted management interventions, including reduction in disturbance. To evaluate whether such interventions could be effective at a mainland protected area in Scotland (St Abb's Head), we analysed a dataset of > 1400 kittiwake nests monitored across nine plots over 7 years (2015–19 and 2022–23) to (a) assess potential impacts of increasing numbers of walkers on cliff tops and boats operating close inshore on nest success and number of chicks fledged, and (b) assess whether productivity is sufficient to sustain the local population. We found no strong evidence that people or boat activity negatively affected nest success or number of chicks fledged. Rather, we observed weak positive correlations between human presence and both nest success and the probability of successful nests fledging more than a single chick, which potentially demonstrates a ‘human shield’ effect reducing predation rates. This suggests that mitigation of disturbance at this colony would not represent a viable compensation measure for kittiwakes. However, productivity (chicks per pair) across the study period was at the lower end of levels previously predicted to sustain kittiwake populations. This raises concerns about the future viability of the population if juvenile or adult survival rates decline due to anthropogenic drivers such as offshore wind development, climate change, or impacts of novel infectious diseases.

## Graphical abstract

Offshore wind energy is rapidly expanding in the North sea foraging grounds of internationally important seabirds, with predicted impacts on their survival and productivity. Since reduction to breeding colony disturbance is a proposed compensation measure for these impacts, we used a seven‐year dataset of > 1400 black‐legged kittiwake nests at an important mainland breeding colony to assess if intensity of people and boat traffic near nest sites caused measurable impacts on their breeding success. We found no negative relationships between people or boat traffic and kittiake breeding success, but instead and a weak positive relationship with successful nests producing more than one chick, suggesting reduction in colony disturbance at this site may not be a suitable compensation measure for impacts of offshore wind farms.

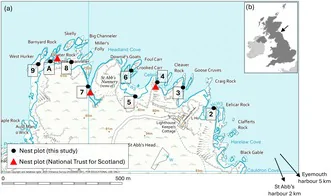

## 1 Introduction

Seabirds breed on land in often highly visible colonies, but most species feed predominately at sea. They are widely regarded as sentinel species for the health of marine ecosystems (Lescroël et al. 2016; Velarde et al. 2019). As indicator species, evidence of declines in their populations have revealed the combined effects of ecosystem changes such as rising sea temperatures, reduced prey availability, fisheries discard bans in some jurisdictions (reducing food availability for many scavenging seabird species) and increased predation by avian and mammalian predators at nesting colonies (Frederiksen et al. 2007; Furness and Tasker 2000; Oro and Furness 2002; Sherley et al. 2020). A global assessment in 2012 found that of 346 seabird species, almost one third were threatened with extinction, 5% were listed in the highest IUCN Red List category of Critically Endangered, and nearly half were known or suspected to be in population decline (Croxall et al. 2012). Over two thirds of seabird species face multiple threats (Dias et al. 2019). Britain supports a globally important population of approximately eight million breeding seabirds from 25 species (JNCC 2024). Populations breeding on the British Isles face multiple pressures, including oil spills, changes in food availability and distribution linked to discard bans, and indirect effects of climate change (Mitchell et al. 2004). More recently, threats such as the expansion of offshore windfarms (Peschko et al. 2020) and outbreaks of high pathogenicity avian influenza (Atkinson and Baillie 2025) have become increasingly significant.

Black‐legged kittiwakes *Rissa tridactyla* (family Laridae; hereafter just ‘kittiwakes’) are pelagic gulls widely distributed across the Pacific, Atlantic and Arctic Oceans. They are categorised internationally as Vulnerable (IUCN 2025) and are on the UK Red List (RSPB et al. 2021). UK breeding numbers declined by 51% between 1986 and 2023 to c. 200,000 breeding pairs (BTO 2025; BTO et al. 2023). Kittiwakes winter across a large area of the North Atlantic, returning to lay eggs in UK colonies in April and May (Carroll et al. 2017). There is a large amount of overlap in wintering areas of birds from breeding colonies in NW Europe, NE Canada and Greenland, but about 85% are thought to winter west of the Mid‐Atlantic Ridge (Frederiksen et al. 2012). Productivity and adult survival are negatively correlated with higher winter sea surface temperatures (Frederiksen et al. 2004), an effect linked to temperature‐driven declines of sandeel populations (Arnott and Ruxton 2002). There is some evidence that kittiwake breeding success at colonies on the east coast of Scotland was depressed due to reduced food availability during a sandeel fishery operation in the period 1990–2000 (Searle et al. 2023). In one Scottish colony, breeding success was higher when sandeels from the current year were larger (Lewis et al. 2001), and in Norway chick growth rates were higher when there was a greater proportion of sandeels in their diet (Christensen‐Dalsgaard et al. 2018). Kittiwakes are particularly susceptible to low sandeel availability because they are surface feeders and can only capture prey within the top few centimetres of the water column (Furness and Tasker 2000). During periods of low food availability, adults spend more time away from the nest, leaving chicks unprotected from predation (Hatch and Hatch 1990), while increased variability in foraging trips and more intensive foraging are associated with lower productivity (Schlener et al. 2024). Although their nesting sites on cliffs are largely inaccessible to terrestrial predators, kittiwake nests are vulnerable to predation by corvids (family Corvidae), raptors (Accipitriformes and Falconiformes), skuas (Stercorariidae) and larger gulls, pressures that are exacerbated when food is scarce (Massaro et al. 2000).

Offshore windfarms are an important component of global strategies to reduce fossil fuel consumption. They also have effects on marine biodiversity, including potential negative impacts such as seabird displacement and mortality (Watson et al. 2025). Kittiwakes may be particularly vulnerable because their typical flight heights overlap with rotor swept zones and their long foraging trips may increase the likelihood of encounters. There is also some evidence that kittiwakes may be attracted to offshore windfarms due to increased food abundance caused by increased primary productivity and aggregations of fishing vessels around windfarms (discussed in Pollock et al. 2024). The first offshore windfarm in the UK was completed in 2000, and the first floating windfarm, allowing generation in deep waters, was commissioned in 2017 (RenewableUK, n.d.). By 2024, UK offshore wind capacity had reached 14.7 GW and, with several large projects in construction or planning phase, capacity is predicted to exceed 40 GW by 2030 (RenewableUK 2025). The spatial footprint of all areas for which the Scottish Government awarded lease options for offshore wind development in 2022 is 9569 km^2^ which is about 5% of Scotland's sea area (Scottish Government 2025a).

Although expansion of offshore wind energy is a stated Scottish Government ambition (Scottish Government 2026a), proposed offshore windfarms in Scottish waters must go through a planning process and be approved by ministers in line with appropriate legislation. Recently, the Scottish Government has developed new policy for environmental compensation for offshore windfarm impacts on protected seabird species. ‘Compensation’ here refers to measures implemented to benefit protected species where a windfarm is predicted to have an adverse impact. Following consultation (Scottish Government 2025b), new regulations (Scottish Government 2026b) and statutory guidance (Scottish Government 2026c) came into force. Prior to these, compensatory measures were normally required to have ‘direct evidentiary links back to the impacted species and/or habitat’ (Scottish Government 2026c). The changes, however, enable the use of compensatory measures that support the UK Marine Protected Area (MPA) network as a whole. These are called Tier 3 measures (previously ‘wider measures’) and can only be used where measures that benefit the impacted feature (Tier 1), or a similar feature (Tier 2), are not available or sufficient, or where Tier 3 will provide a greater ecological benefit. Because opportunities for Tier 1 or Tier 2 compensation are likely to be limited (see Scottish Government 2025b), there is a need for evidence‐based, quantifiable Tier 3 measures. An example of a proposed wider/Tier 3 measure is the reduction of disturbance at breeding colonies (Scottish Government 2025b).

Participation in nature‐based sports, encompassing both land‐ and water‐based outdoor activities, has increased across much of the Global North, with a growing diversity of participants (Melo et al. 2020). In the UK, the number of individual participations in outdoor‐related activities rose from 1.2 billion to 1.5 billion between 2011 and 2016 (Office for National Statistics 2021). Outdoor recreation can provide substantial health and well‐being benefits to people (Thompson Coon et al. 2011), and ecotourism has the potential to benefit local communities and the economic sustainability of marine protected areas (Casimiro et al. 2023). However, increased visitation to coastal areas, whether on foot or by boat, may generate negative direct or indirect impacts on wildlife and biodiversity, including already under‐pressure seabird breeding and foraging populations.

Nisbet (2000) defined disturbance at breeding colonies as ‘any human activity that changes the contemporaneous behaviour or physiology of one or more individuals within a breeding colony’. Behavioural responses may divert time and energy from fitness‐enhancing activities such as foraging or parental care (Rees et al. 2005; Shealer and Haverland 2000; Thomas et al. 2003). Beale and Monaghan (2004) found that nesting success of guillemot *Uria aalge* and kittiwake decreased with increasing cliff‐top visitor numbers and decreasing distance between nests and visitors. Modelling suggested that this response was driven by perceived predation risk. Tourist access to yellow‐eyed penguin *Megadyptes antipodes* reduced chick survival, with chicks in high‐tourist areas weighing significantly less than those in tourist‐free areas (McClung et al. 2004). De Villiers et al. (2006) showed that heart rates of northern giant petrels *Macronectes halli* increased by up to 204% over resting levels when people were up to 40 m away. Burger (1981) found that even limited human disturbance, such as an investigator walking near a breeding colony for 30 min every other day, can reduce breeding success of herring gulls *Larus argentatus* and great black‐backed gulls *Larus marinus* .

Boats may also impact seabirds at nest sites and while foraging. Boat disturbance can strongly alter common tern *Sterna hirundo* flight behaviour, particularly during early breeding stages; approaching boats have been shown to cause up to 500 terns to take flight above a colony and remain airborne for up to 3 min after departure, potentially increasing energetic costs and exposing nests to predation and chilling (Burger 1998). Foraging distance from shore, distance from boat, and boat speed all significantly increased the likelihood of flushing in black guillemots *Cepphus grylle* (Ronconi and Clair 2002). European shags *Phalacrocorax aristotelis* exhibited boat‐avoidance behaviour in a marine protected area (MPA), with foraging activity decreasing with boating levels, and some feeding areas being excluded from use (Velando and Munilla 2011). Rojek et al. (2007) reported that disturbance‐related behaviours of guillemots (head‐bobbing, movements or flushing) occurred predominantly when vessels were within 50 m of nests and remained in the area for extended periods. Higher levels of boat disturbance were linked with increased loss of eggs and chicks, likely caused by flushing or nest abandonment.

Given the Red List status of kittiwakes in the UK (RSPB et al. 2021) and their global classification as Vulnerable (IUCN 2025), combined with increasing pressures from offshore wind energy development, this study aimed to: (a) assess the potential effects of people and boats near nesting cliffs on nest success and number of chicks fledged at a nationally important mainland breeding colony and protected area experiencing increasing visitor numbers; (b) quantify kittiwake breeding success to determine whether productivity was sufficient to sustain the population under assumed demographic rates and thus assess vulnerability of the population to demographic change; and (c) assess the degree of evidence for anthropogenic disturbance‐related impacts on kittiwake breeding to inform the potential use of disturbance mitigation as a compensatory measure for offshore windfarm impacts.

## 2 Methods

### 2.1 Study Site

The study was conducted at St Abb's Head National Nature Reserve in the Scottish Borders region, UK. Managed by the National Trust for Scotland (NTS), the site is designated as a Site of Special Scientific Interest (SSSI) and Special Protected Area (SPA) for its breeding seabirds including common guillemot, razorbill *Alca torda* , northern fulmar *Fulmarus glacialis* , and kittiwake, with a 1‐km marine extension to the SPA classified in 2009 (Scottish Natural Heritage 2009). Whole‐colony counts of ‘Apparently Occupied Nests’ (AONs) of kittiwakes and other species have been conducted sporadically between 1957 and 1985 and then annually thereafter. AONs are defined as ‘well‐constructed nests, attended by at least one adult and capable of holding an egg (even if nest contents are unknown, or the nest is known to be empty)’ (Walsh et al. 1995). Kittiwake numbers peaked at 19,066 AONs in 1989, declined through the 1990s and 2000s and during the study period (2015–2023) averaged 4447 AONs (range 2779–5851). The kittiwake's decline from the late 1980s at St Abb's Head appears to mirror national (UK) declines. The species' population increased by 24% between national seabird censuses in 1969–70 and 1985–88 (BTO 2025), but systematic manual monitoring since then has shown declines in Scotland, Wales and England (Harris et al. 2024). The causes are thought to relate primarily to reductions in food availability, driven by (i) changes in sea surface temperature modifying the distribution and development of their main prey lesser sandeels *Ammodytes tobianus* , competition from sandeel fisheries, and (ii) climate change making heavy summer rainfall and winter storms more common, influencing both winter feeding and summer breeding (Harris et al. 2024).

An automated people counter at the reserve entrance, near the main site carpark and visitor centre, shows that annual visitors by foot have risen from c. 60,000 in the mid‐2010s when the present study started, to c. 100,000 in 2024/25. However, these figures do not indicate how visitors distribute their time within the reserve, and an alternative, albeit less frequently used, entry point is present but uncounted. While boat numbers have not been systematically recorded, observations by reserve rangers suggest that vessel visits close inshore to the colony have increased over the past decade. The motivations for boat visits are described below.

### 2.2 Study Design

**FIGURE 1:**
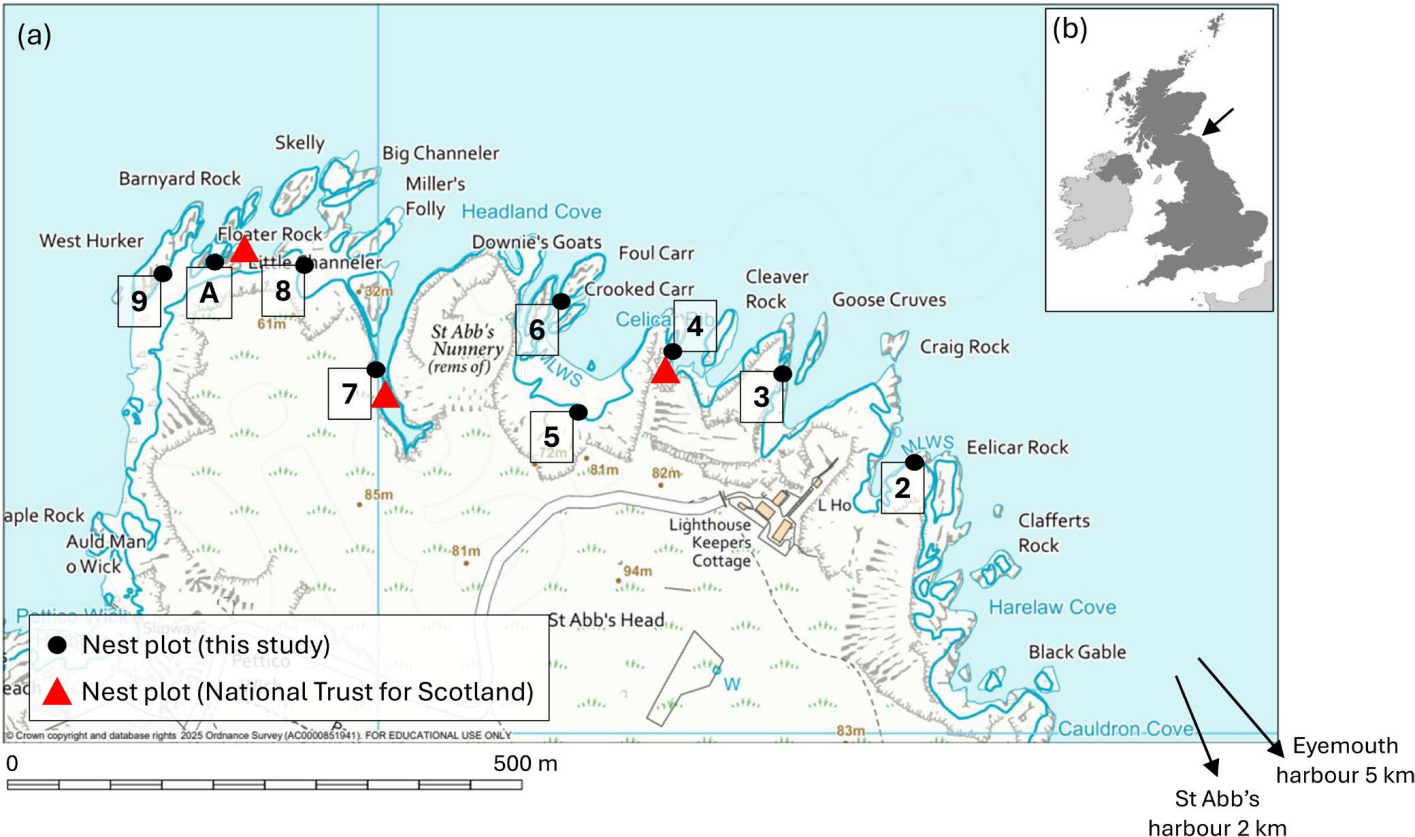
St Abb's Head National Nature Reserve study site: (a) locations of nine nest plots (2–9 and A) monitored at 2015–19 and 2022–23 in this study and three long National Trust for Scotland long‐term monitoring plots; and (b) location of this mainland colony on Great Britain. Shown are the approximate distances to the two main harbours where boats (including diving, fishing, power boats and kayaks) visit the site from. UK outline CC BY‐SA 3.0.

Most kittiwake nests at the site are located along a series of cliffs and stacks approximately 800 m to 1 km in length, broadly facing NNE (Figure 1). From the seaward side, the coastline forms a series of complex coves that differ in their appeal and accessibility to various boat users (discussed below). On land, most people approach either from the visitor centre about 1.5 km from the study area or via a small carpark by a lighthouse situated towards the east end of the study site. This access pattern was assumed to generate natural variation in exposure to both people and boats along the colony. The site is fully accessible and, in line with Scottish outdoor access legislation (NatureScot 2025), there are no restrictions placed on how close people can approach the edge of the cliffs, nor are there any fixed restrictions on how close boats can access coves. To capture variation in these two crossed factors, while maintaining some independence between sampling plots, we selected nine relatively evenly spaced ‘plots’ along the length of the headland. A plot is defined as a concentration of kittiwake nest sites that could be readily identified and relocated between years. Data were collected during the breeding seasons of 2015–19 and 2022–23. No data could be collected in 2020 or 2021 due to the Covid‐19 pandemic and fieldworker restrictions.

Plots were originally numbered 1 to 9 from east to west. Plot 1 was on a smaller headland separated from the others by a bay, several hundred metres to the east (not shown in Figure 1). This plot was dropped after the first year and replaced with a new plot A, located between Plots 8 and 9. Plots 2–9 and A were monitored in all years, except for plot A in 2015 (not yet established) and in 2022 due to fieldworker safety concerns related to the steep viewing angle.

Apart from some data collected by the ranger team (discussed below), most field data were collected by seven of the authors conducting their master's degree research project, each in a separate year. This provided the students with ornithology field skills and the opportunity to experience working with a protected area ranger team, while allowing the collection of data across multiple years. The process of nest recording was standardised throughout the seven study years via: (1) detailed instructions on nest recording and data entry, and (2) in several years the previous year's fieldworker visited during the first week of data collection to help standardise the approach. However, some flexibility was retained to allow students to design (and ‘own’) elements of their research, resulting in some year‐to‐year variation in data collection relating to people, boats and physical nest site characteristics (discussed below). Laying dates in kittiwakes vary annually but at St Abb's Head the first laying date over the course of our study from the three separate plots monitored by National Trust for Scotland (details below) was 20 May (range 15–28 May from all study years except 2017 for which data were not available). Due to interannual variation in teaching schedules, master's students' start dates varied between years (mean 24 May, earliest 18 May in 2018 and latest 5 Jun in the first study year). Thus, some very early nest failures may not have been recorded. Differences in start dates and in fieldworker approaches to recording people and boat activity were accounted for in the analyses below.

Field data were collected by site visits typically carried out 3 days per week from May to August. Each week, the fieldworker aimed to collect nest data, people data and boat data from each plot once. This was determined to be the most feasible sampling frequency. Nest monitoring took longest in early incubation when adults sit tightly on eggs, and it was only possible to monitor three plots per day at this stage. Consequently, all nine plots could be surveyed over 3 days. This monitoring was almost always conducted from Friday to Sunday to coincide with peak visitor and boat activity on these days, thereby capturing conditions of maximum potential disturbance. To avoid potential bias from variation in people or boat activity between or within days, the order in which plots were monitored was rotated within each year so that each plot was observed across a range of days (Friday, Saturday, Sunday), and time of day (early, middle, late). Occasionally, adverse weather forced surveying on alternative days, but this impacted only a small proportion of weekends across the study.

### 2.3 Nest Monitoring

Nest monitoring followed the JNCC Seabird Handbook for Britain and Ireland kittiwake productivity‐monitoring methods (Walsh et al. 1995) but was adapted to use the daily survival rate method (often referred to as the ‘Mayfield Method’) (Mayfield 1975). This approach is commonly applied to non‐colonial species where not all nesting attempts can be detected, such as waders (Jarrett et al. 2024) and passerines (Matysioková and Remeš 2022). The daily survival rate method is based on recording ‘exposure days’ per nest (days within the period that the nest was being monitored and was active and thus exposed to potential failure). For successful nests, exposure days and ‘success days’ are equal, whereas for failed nests, success days are one fewer than the exposure days. We applied this method to kittiwakes to (i) counter potential bias arising from very early nest failures that may have gone undetected due to variation in fieldwork start dates, (ii) allow incorporation of new nests added as the season progressed and monitoring time demands decreased (adults sit less tightly on nests later in the season). We subsequently assessed this approach by comparing our productivity estimates with those derived by NTS from nearby plots using a more widely applied method, and by examining the presence of any systematic bias, described below.

To obtain exposure days and success days, at each visit we recorded whether each nest was active or not. Given the weekly visits, first egg dates (start of nesting) and failure dates (where applicable) were calculated using the midpoint assumption, rounding forward by 1 day if the interval between visits was an odd number of days (Hazler 2004). Failure was assumed if the nest became empty at any point before the chicks reached the ‘large’ stage (Walsh et al. 1995). If a nest was added to the sample after it had already started, exposure days were only taken from the date the monitoring of that nest began. Because kittiwake chicks may leave and return to the nest (or an adjacent nest) when at an advanced development stage, fledging cannot be determined as definitely as in some other bird species where leaving the nest is effectively a single, irreversible event. Therefore, if chicks were recorded as ‘ready to fledge’ during monitoring, this was considered a successful fledge and that visit was marked as the fledge date. Images of what we considered as ‘ready to fledge are in Appendix A. The apparent number of chicks fledged per successful nest was also recorded for each nest.

### 2.4 Nest Site and Plot Characteristic Variables

Two physical nest site characteristic variables were recorded based on methods utilised in Regehr et al. (1998), namely ‘overhang’ and’ underjut’. Overhang was defined as the presence of a solid projection protruding from the cliff wall above a nest, providing some potential protection from adverse weather and overhead predation. Underjut was defined as a solid projection protruding underneath or adjacent to a nest acting as a potential perch for avian predators. Three plot‐level characteristics were recorded based on methods by Beale and Monaghan (2004). These were the ‘height’ of the centre of the plot in relation to the water (to the nearest 5 m), what we call ‘dip’ which was the distance from the centre of the plot to the nearest edge of the landmass (i.e., where a human could stand closest to the plot, often above it; to the nearest 5 m) and whether the plot was ‘onshore’ (i.e., on a cliff contiguous to the mainland) or not (located on a stack that was separated by any water at low tide).

### 2.5 Monitoring People and Boats

Note we use the term ‘people’ to refer to visitors on land on the cliff tops. The general approach for estimating potential disturbance from people was to record an index of people presence within a zone that was visible to a given plot. We estimated this zone based on a person walking along the cliff top and seeing which areas of the cliff top the centre of the nest plot was visible from. The assumption was that if the plot was visible from a given point on the cliff top, a person at that location would also be visible to at least some birds within the plot. We did not include the single observer in the count as this was consistent across all sites within and between years. The closest that people could approach to nests at any one plot was only a few metres (the centre of Plot 4 was only 5 m from the cliff top). However, we did not measure how close people were to the plot as this would represent a challenging three‐dimensional measurement due to the complexity of the topography. In addition, for some plots, where people would be closest to the plot (e.g., above it), they were not likely to be visible from the nests below. However, distance to cliff top was captured in the variable ‘dip’ described above.

Estimating potential boat disturbance was more challenging as we did not have access to a boat to conduct an equivalent visibility assessment. As such, we recorded only boats present within the immediate foreshore adjacent to a plot, that is those engaged in activities related to the cliffs, such as dropping off scuba divers, showing tourists the cliffs, sea kayaks investigating the coves etc. This distinction was generally clear, as boats simply passing the headland tended to be further offshore. In this study we did not directly measure the distance of boats to the kittiwake nest plots. However, in a later study relating to guillemot behavioural responses to boats in 2024 and 2025, where we used a laser range finder at sites close to plots A and 4 in this study (unpublished data), both unpowered (e.g., kayaks) and powered (e.g., rigid inflatable) boats approached the foot of the cliffs to within touching distance.

For both boats and people, we adopted the approach of a 30‐min sampling period per plot per week. Monitoring typically spanned 12–14 weeks per year, representing 6–7 h of seasonal sampling per plot, rotated across different days (Friday–Sunday) and times of day (early, middle, late). We calculated ‘people minutes’ and ‘boat minutes’ as the cumulative time that people or boats were within sight of a plot during the sampling period. To account for variation in the number of monitoring weeks between years, these values were standardised by dividing by total monitoring hours, producing ‘people minutes per hour’ and ‘boat minutes per hour’. Boat observations were further subcategorised as ‘dive boat minutes per hour’, ‘power boat minutes per hour’, ‘fishing boat minutes per hour’ and ‘kayak minutes per hour’. Dive boats were motorised vessels distinguished by behaviour such as dropping off divers and frequently waiting in a cove or nearby, often with the engine running. The activity of dive boats could include use of noisy elevators to lower divers in and out of the water and shouting between divers in the water and boat operators. Power boats were typically rigid inflatable boats (RIBs) used for tourism or lifeboat training. Fishing boats were typically ‘creel boats’ placing or retrieving creels (also known as ‘lobster pots'). Both power and fishing boats generate substantial noise above and below water.

### 2.6 Data Variation and Processing

**FIGURE 2:**
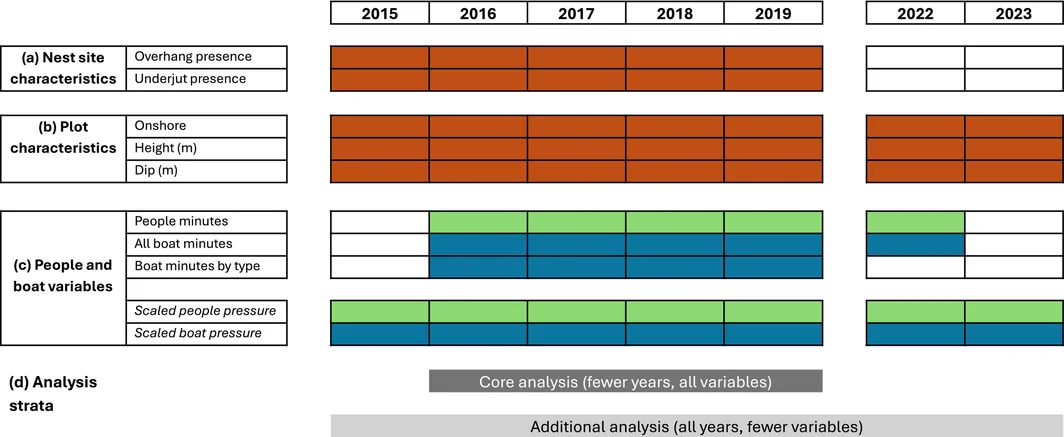
Data availability by year and corresponding analyses. Coloured cells indicate the collection of a variable or method, while white cells indicate no collection that year. Brown colouring denotes nest site or plot characteristics, green indicates people‐related variables, and blue indicates boat‐related variables.

To minimise errors in the data (1458 rows), summaries were generated for each variable and any extreme values double checked and removed where there appeared to be mistakes (e.g., an unused factor level, an extreme unrealistic value etc.). As mentioned above, while nest monitoring methods were standardised, some differences in data collection occurred between fieldworkers and years. The availability of different predictor variables by year is summarised in Figure 2. Nest site characteristics (Figure 2a) required precise location data of numbered nest and were not collected in 2022–2023 (i.e., after the break in study years due to Covid‐19 impacts). In contrast, plot characteristics remained constant between years and were therefore available throughout the entire study period (Figure 2b). The recording approach for people and boat variables varied between years (Figure 2c). The variables of ‘people minutes per hour’ and ‘boat minutes per hour’, as described above, were collected from 2015 to 2022, but raw data from 2015 were unavailable and standardisation could not be guaranteed, so these were excluded. In 2023, data on people and boats were collected using an alternative approach based on a ‘number of human disturbances’ and ‘boat passes’ per hour, not directly comparable to people minutes per hour and boat minutes per hour. Finally, versions of boat minutes per hour separated by boat type (dive boats, fishing boats, power boats and kayaks) were not collected in 2022, only for all boat types combined.

For these reasons, we identified two strata in our data (Figure 2d): (i) a ‘core analysis’ stratum comprising 4 years (2015–2019), in which all variables were collected in a fully standardised way, and (ii) an ‘additional analysis’ stratum including all seven study years (2015–19 and 2022–23) in which some variables, such as nest site characteristics and certain measures of people and boat pressure, were missing or collected inconsistently.

To account for differences across variables, for the ‘additional analysis’ stratum we calculated ‘scaled people pressure’ (SPP) and ‘scaled boat pressure’ (SBP) variables for each plot in each year. These are explained in Appendix B.

### 2.7 Assessment of People and Boat Impacts on Nest Success

Analyses were carried out in R (R Core Team 2023) and RStudio (RStudio Team 2020), using the lme4 (Bates et al. 2015) and MuMIn (Bartoń 2020) packages. Modelling was conducted using generalised linear mixed models (GLMM) with binomial error distribution and logit link function.

For nest success an adaptation of the Mayfield Method within a generalised linear model framework was used (Aebischer 1999). The response variable comprised success days as the binomial numerator, and exposure days as the denominator. Model predictions were made on the logit scale and after back transformation these represent daily survival probability (DSP), which is the probability a nest will survive 1 day within the nest cycle. All variables using in modelling are shown in Table 1.

**TABLE 1:** Summary of variables used in modelling daily survival probability of nests, or in subsequent calculations of nest success and seasonal productivity.

Group	Variable	Abbreviation	Type	Scale
Nest success sub‐variables	Exposure days	total.expos	Integer	Occupied nest site in year
	Success	success	Binary	Occupied nest site in year
Site	Overhang presence	overhang	Binary	Nest site
	Underjut presence	underjut	Binary	Nest site
Plot	Onshore	onshore	Binary	Plot
	Height (m)	height	Continuous	Plot
	Dip (m)	dip	Continuous	Plot
People and boat variables	People minutes	people.mins	Continuous	Plot in year
	Scaled people pressure	people.scaled	Continuous	Plot in year
	Boat minutes	boat.mins	Continuous	Plot in year
	Dive boat minutes	dive.mins	Continuous	Plot in year
	Power boat minutes	power.mins	Continuous	Plot in year
	Fishing boat minutes	fishing.mins	Continuous	Plot in year
	Kayak minutes	kayak.mins	Continuous	Plot in year
	Scaled boat pressure	boat.scaled	Continuous	Plot in year
Random effects	Plot	plot	Categorical	—
	Year	year	Categorical	—

Nests fledging three chicks were extremely rare (0.4%, *n* = 4 across all years), with most successful nests fledging either one (58%, *n* = 531) or two chicks (41%, *n* = 378). The response variable of chicks per successful nest was therefore treated as binary (one chick vs. more than one chick fledged). Model predictions were made on the logit scale and back‐transformed to give the probability of a successful nest fledging at least two chicks.

To explore potential relationships between people, boats and nest success, we developed a set of candidate models, which differed between the core analysis (2016–2019; 72 models) and the additional analysis (2015–2019 and 2022–2023; 16 models) due to different number and types of available variables (Table 2). All models contained year and plot as crossed random effects to account for non‐independence within years or plots, coded as random intercepts. For the additional analysis, we also included random slopes (Harrison et al. 2018) for scaled people pressure and scaled boat pressure to account for different measurement approaches but the same underlying hypothesis of a directional effect. Interactions between variables were not tested due to frequent convergence issues. We also built models with year as a linear fixed effect to account for any general trends in nest success or output per successful nest, but it produced a high incidence of singular fit or convergence issues, which were not an issue in models without it.

**TABLE 2:** Candidate model sets including fixed and random effects for core and additional (‘addit.’) analyses (see Figure 2). For brevity, variable groups are shown in square brackets. Individual variables within each group are listed in Table 1.

Model set	Number of models			Random effects	
	Core	Addit.	Fixed effects	Intercepts	Slopes
Null	1	1	none	year + plot	
Site	2		[site variable]	year + plot	
Plot	3	3	[plot variable]	year + plot	
People	1		people.mins	year + plot	
		1	people.scaled	year + plot	people.scaled|year
Boats	1		boat.mins	year + plot	
	4		[boat type variable]	year + plot	
		1	boat.scaled	year + plot	boat.scaled|year
Combined	5		[site or plot variable] + people.mins	year + plot	
		3	[plot variable] + people.scaled	year + plot	people.scaled|year
	5		[site or plot variable] + boat.mins	year + plot	
	20		[site or plot variable] + [boat type variable]	year + plot	
		3	[plot variable] + boat.scaled	year + plot	boat.scaled|year
	1		people.mins + boat.mins	year + plot	
	4		people.mins + [boat type variable]	year + plot	
		1	people.scaled + boat.scaled	year + plot	people.scaled|year + boat.scaled|year
	5		[site or plot variable] + people.mins + boat.mins	year + plot	
	20		[site or plot variable] + people.mins + [boat type variable]	year + plot	
		3	[plot variable] + people.scaled + boat.scaled	year + plot	people.scaled|year + boat.scaled|year
Total	72	16			

We used the small‐sample corrected Akaike's Information Criterion (AIC_c_) to rank the candidate models, with inference drawn from models with Δ_AICc_ of < 2 (Burnham and Anderson 1998). Model‐averaged parameter estimates and standard errors, weighted by each model's Akaike's weight (*w*_*i*_), were calculated to account for model uncertainty (Johnson and Omland 2004).

### 2.8 Annual Productivity and Population Sustainability

Coulson (2017) estimated that maintaining kittiwake breeding numbers in Britain requires a mean productivity of 0.8–1.5 chicks per pair. We therefore compared our annual productivity estimates over the course of our study to that range. Daily survival probability (DSP) estimates of nests were translated to annual productivity estimates (chicks fledged per pair) using the following procedure. First, we estimated nest success (the probability of nest fledging at least one chick) using the equation:

(1) $${\text{nest success}}_{\text{year}}={{\mathrm{DSP}}_{\text{year}}}^{d}$$

where DSP_year_ is the estimated annual daily survival probability and *d* is the duration of a successful nest. DSP_year_ was estimated by running a null GLMM with ‘plot’ as a random effect, separately for each year. We obtained *d* by summing assumed laying, incubation and provisioning periods. The incubation period was calculated as the mid‐point (rounded up to nearest whole day) between the minimum and maximum reported incubation lengths for kittiwakes, 25 and 32 days, respectively (BTO 2025). With the assumption that two eggs are laid with a day's gap between, a laying period of 3 days was added to account for the fact that incubation only commences after the final egg has been laid (Threlfall and Maunder 1972), giving a total of 32 days. These externally‐sourced values were used because laying dates could not be reliably estimated in our study, and incubation duration is likely to be more physiologically constrained and less variable.

The provisioning period, on the other hand, appears to be more variable, with BTO (2025) reporting 33–54 days. Thus, we estimated the provisioning period from our own data by selecting all successful nests for which both estimated hatching and fledging dates were available, ensuring the entire provisioning period was monitored. A null GLMM with ‘year’ and ‘plot’ as random effects was used to take the estimated provision period, yielding 35.1 (±1.1 SE) days, which falls within the range provided by BTO (2025). Accordingly, *d* was set to 32 days (laying and incubation) + 35 days (provisioning) = 67 days.

To translate nest success into an estimate of annual productivity (chicks per pair) we used the equation:

(2) $$\text{chicks}\;\mathrm{per}\;\text{pair}={\text{nest success}}_{\text{year}}\times {\mathrm{CSN}}_{\text{year}}$$

where CSN_year_ is the estimated number of chicks fledged per successful nest for a given year. This was recorded by our field workers, and to account for different sample sizes between plots, we used a null GLMM with year and plot as random effects. For 2022, the raw data were not available for this variable, so we substituted it with the all‐years mean value derived from a null GLMM with year and plot as random effects, noting this limitation in subsequent interpretation.

Staff at NTS, the land manager, had collected kittiwake productivity data (chicks per nest) annually from three separate plots at the site, using the standard Seabird Monitoring Handbook approach (Walsh et al. 1995) as part of their long‐term monitoring. This approach estimates productivity by dividing the number of chicks determined to have fledged across a sampling plot by the number of ‘completed nests’ (a nest that is fully built by a pair in that year, regardless of its later success or failure). These plots were close to some of our plots (Figure 1) but did not overlap. These data were not used for our study as their method focussed on fewer plots but containing more nest sites per plot, whereas to sample sufficient variation in people and boats across plots, we adopted a strategy of more plots but fewer nest sites per plot. Nonetheless, the NTS data provided an opportunity to compare our nest success estimates based on the Mayfield Method to those data derived from the alternative approach. The annual number of nests monitored was similar between sources, although sample sizes from NTS were more consistent between years (208 ± 67 SD pa in our study, 193 ± 23 SD pa from NTS). We assessed potential systematic bias via a scatterplot including a line of equal value between methods (*x* = *y*). Systematic bias in either method relative to the other would be indicated by points lying predominantly above or below that line. As no such systematic bias was detected (see Section 3), we collated our data with the NTS dataset to produce overall annual productivity estimates for St Abb's Head LNR, thereby maximising sample size. Weighted averages were calculated by multiplying each method's annual productivity estimate by its sample size and dividing by the total sample size across both methods. We then plotted the weighted annual productivity estimates, against Coulson's (2017) range of 0.8–1.5 chicks per pair.

## 3 Results

### 3.1 Summary of Daily Survival Probability, People and Boats

Over the seven‐year study period, 1458 kittiwake nests were monitored across our nine plots. A median of 257 nests was surveyed per year summed across all plots (range 95–272), with a median of 170 nests per plot summed across all years (range 100–195). The median number of nests monitored per plot per year was 25 (range 12–51). Across the study, 140 nests were monitored during incubation only, 503 during chick rearing only, and 815 during both incubation and chick rearing. There was a significant interaction between plot and year (as fixed effects) on daily survival probability ($\chi_{46}^{2}$ = 104.7, *p* < 0.001), demonstrating variation across years and plots. Across all years, estimated daily survival probability of nests varied between 0.991 and 0.996, equivalent to nest success as low as 0.56 (2016) and as high as 0.75 (2023) (Table 3a). Annual estimates of chicks fledged per successful nest varied between 1.32 (2019) and 1.60 (2015).

**TABLE 3:** Annual summaries of: (a) nest success and productivity from this study (nine smaller plots) using the Mayfield Method, (b) productivity from the National Trust for Scotland (three larger plots) using the Seabird Monitoring Handbook method and (c) weighted mean productivity estimate (by sample size) combining the two approaches.

	Variable	2015	2016	2017	2018	2019	2022	2023
(a) Nine smaller plots (this study)	Daily survival probability	0.994 (0.990–0.996)	0.991 (0.988–0.994)	0.994 (0.992–0.996)	0.994 (0.990–0.997)	0.993 (0.992–0.995)	0.995 (0.991–0.997)	0.996 (0.992–0.998)
	Nest success *a*	0.65 (0.50–0.76)	0.56 (0.44–0.66)	0.68 (0.60–0.76)	0.69 (0.52–0.81)	0.64 (0.57–0.70)	0.70 (0.54–0.81)	0.75 (0.58–0.86)
	Chicks per successful nest *b*	1.60 (1.48–1.72)	1.54 (1.43–1.66)	1.40 (1.31–1.49)	1.37 (1.27–1.47)	1.32 (1.22–1.41)	1.43* (1.34–1.52)	1.40 (1.31–1.49)
	Productivity *ab* (chicks per pair)	1.04	0.86	0.95	0.95	0.84	1.00	1.05
	Sample size *n*	95	267	259	139	272	257	169
(b) Three larger plots (NTS)	Productivity	1.09	0.77	1.02	0.83	0.86	0.89	0.79
	Sample size *n*	237	178	199	172	179	213	175
(c) Weighted productivity estimate	Productivity (chicks per pair)	1.08	0.82	0.98	0.88	0.85	0.95	0.92

*Note:* Values in parentheses are standard errors.

* Chicks fledged per pair was not recorded in 2022; the value shown is the estimate across all years, accounting for year and plot.

**FIGURE 3:**
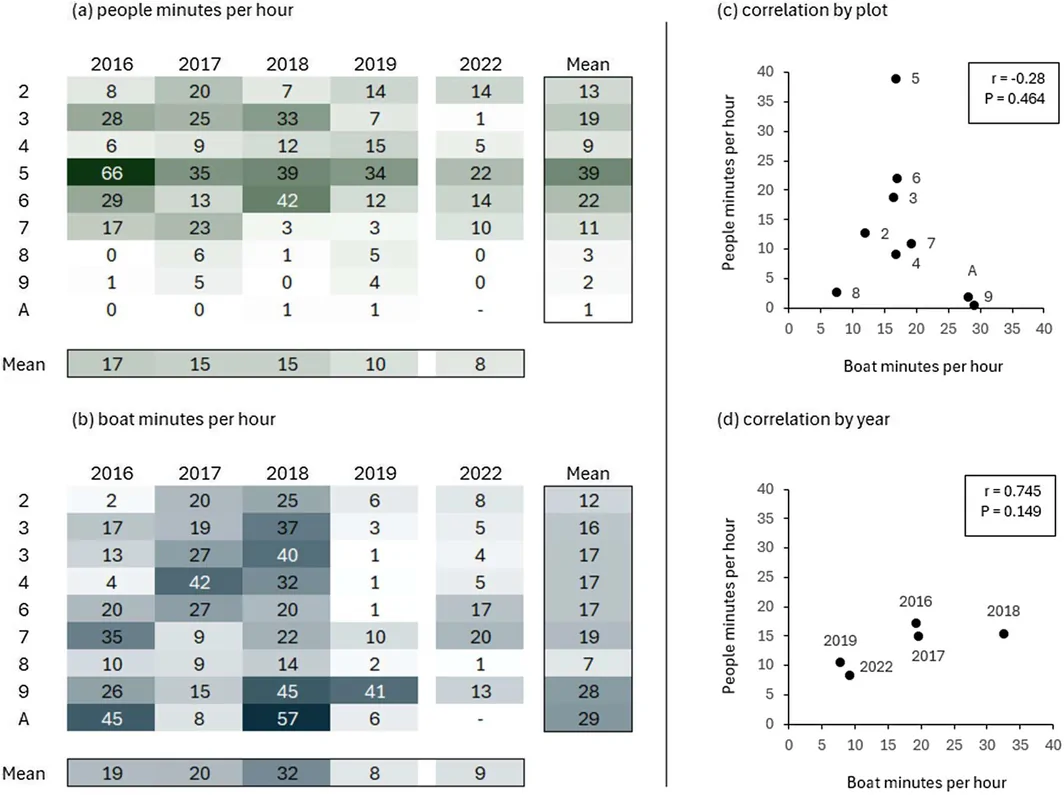
Summary of (a, b) and relationships between (c, d) people and boat minutes per hour from standardised watches. In (a) and (c), each row is a separate nest plot. Correlations by plot and by year are shown separately.

For the 5 years in which people minutes per hour was available as a consistent variable (2016–19 and 2022), values ranged from 0 to 66 per plot per year (Figure 3a), with a mean of 13.0. Similarly, boat minutes per hour varied between 0.5 and 57.0 per plot per year, with a mean of 17.8 (Figure 3b). For the years in which boat type was recorded (2016–2019), dive boats accounted for the greatest mean presence at 12.1 min/h (range 0.0–34.5), followed by fishing boats (2.4 min/h; range 0.0–13.1), kayaks (1.3 min/h; range 0.0–15.0) and power boats (1.1 min/h; range 0.0–8.7). People minutes per hour and boat minutes per hour did not correlate across plots (Figure 3c) or across years (Figure 3d), although the latter comparison was based on a small sample size.

### 3.2 Effects of Walkers and Boats on Nest Success and Output

There were no clearly supported models in our analysis of nest success for either the core or the full dataset (Table 4). In both cases, the null model was within Δ_AICc_ ≤ 2 of the top‐ranked model and the best models had low Akaike weights (*w*_*i*_) of 0.07 and 0.13, respectively. For the number of chicks fledged per successful nest, two models fell within the confidence set (Δ_AICc_ ≤ 2) in the core analysis, although both had relatively low Akaike weights (*w*_*i*_ = 0.21 and 0.10). Both models included people minutes and power boat minutes as predictors, and the top‐ranked model additionally included plot height. In the additional analysis, only a single model was retained within the confidence set, which included plot height alone (*w*_*i*_ = 0.40).

**TABLE 4:** Model selection tables for the analysis of daily survival probability and chicks fledged per successful nest of kittiwake nests for: (a, c) core analysis and (b, d) additional analysis.

Model	Fixed effects	df	−ln(*L*)	AIC_c_	Δ_AICc_	*w*_*i*_
(a) Daily survival probability of nests, core analysis (fewer years, all variables)						
24	underjut + dive.boat.mins	5	−656.8	1323.58	0.00	0.07
3	underjut	4	−657.8	1323.72	0.14	0.06
19	underjut + boat.mins	5	−656.8	1323.76	0.17	0.06
39	underjut + kayak.mins	5	−657.3	1324.63	1.05	0.04
14	underjut + people.mins	5	−657.4	1324.87	1.28	0.03
54	underjut + people.mins + dive.boat.mins	6	−656.4	1324.91	1.33	0.03
49	underjut + people.mins + boat.mins	6	−656.4	1324.95	1.37	0.03
34	underjut + fishing.boat.mins	5	−657.5	1324.97	1.38	0.03
9	dive.boat.mins	4	−658.7	1325.40	1.81	0.03
69	underjut + people.mins + kayak.mins	6	−656.7	1325.40	1.82	0.03
1	*null*	3	−659.7	1325.48	1.89	0.03
29	underjut + power.boat.mins	5	−657.7	1325.56	1.97	0.02
*… 60 more models*						
(b) Daily survival probability of nests, additional analysis (all years, fewer variables)						
6	boat.scaled	6	−1038.7	2089.52	0.00	0.13
5	people.scaled	6	−1038.9	2089.86	0.34	0.11
10	height + boat.scaled	7	−1038.2	2090.54	1.02	0.08
8	dip + people.scaled	7	−1038.3	2090.64	1.12	0.07
13	people.scaled + boat.scaled	10	−1035.3	2090.67	1.15	0.07
9	offshore + people.scaled	7	−1038.3	2090.71	1.19	0.07
12	offshore + boat.scaled	7	−1038.4	2090.83	1.31	0.07
1	*null*	3	−1042.6	2091.23	1.71	0.06
11	dip + boat.scaled	7	−1038.6	2091.25	1.73	0.05
2	height	4	−1041.6	2091.27	1.75	0.05
15	dip + people.scaled + boat.scaled	11	−1034.6	2091.31	1.80	0.05
7	height + people.scaled	7	−1038.7	2091.47	1.95	0.05
*… 4 more models*						
(c) Chicks fledged per successful nest (1 vs. ≥ 2), core analysis (fewer years, all variables)						
60	height + people.mins + power.boat.mins	6	−457.7	927.48	0.00	0.21
45	people.mins + power.boat.mins	5	−459.5	929.11	1.62	0.10
*… 70 more models*						
(d) Chicks fledged per successful nest (1 vs. ≥ 2), additional analysis (all years, fewer variables)						
2	height	4	−599.0	1206.02	0.00	0.40
*… 15 more models*						

*Note:* Random effects are not shown for brevity but presented in Table 2. For brevity, only models with Δ_AICc_ ≤ 2 are shown.

Abbreviations: Δ_AICc_ = difference in AIC_c_ of the top‐ranked model (lowest value) and a given model; AIC_c_ = small sample Akaike's Information Criterion; df = model degrees of freedom; −ln(*L*) = negative log‐likelihood; *w*_*i*_ = Akaike's weight of model.

**FIGURE 4:**
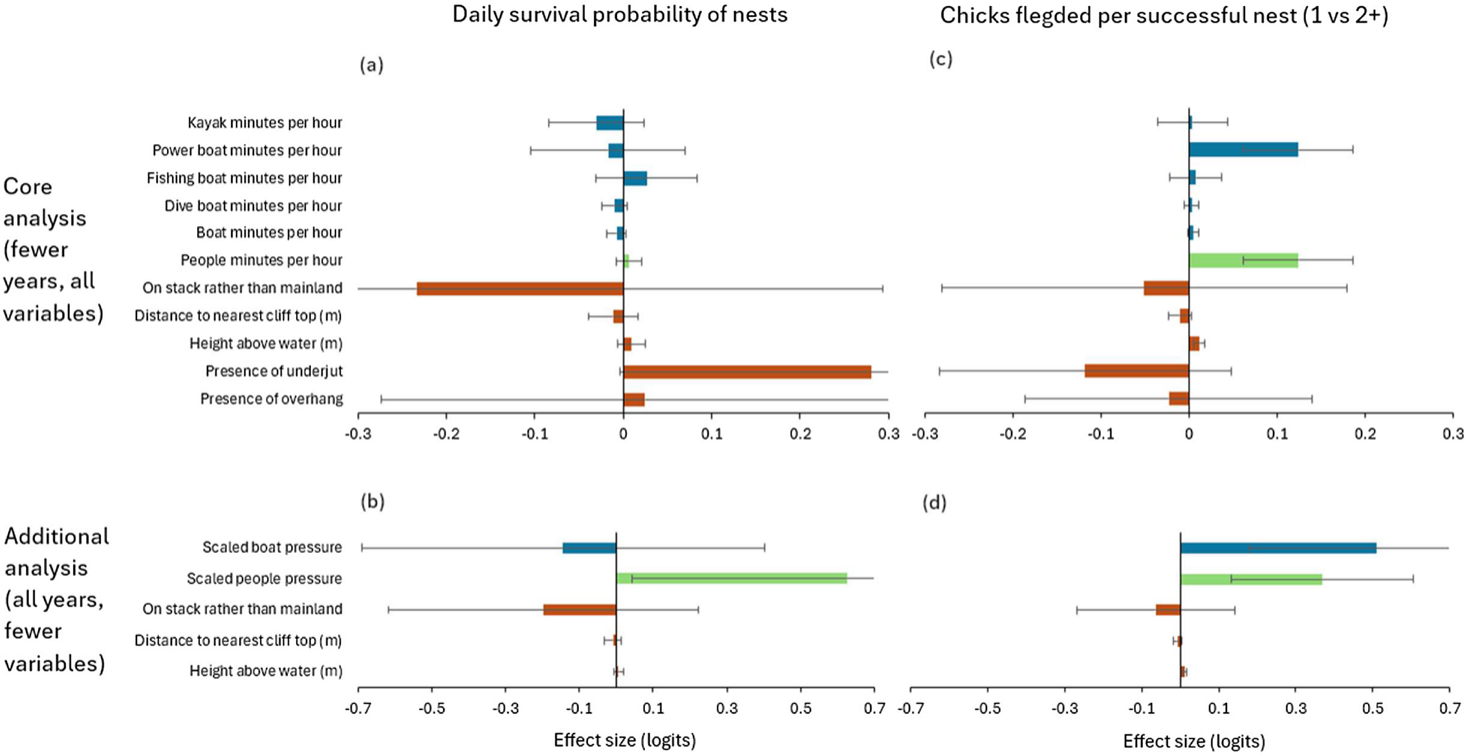
Model‐averaged parameter estimates from model sets summarised in Table 2 including 95% confidence intervals. Brown indicates nest site or plot characteristics, green people‐related variables, and blue boat‐related variables. Note some confidence intervals extend beyond the plot windows.

**FIGURE 5:**
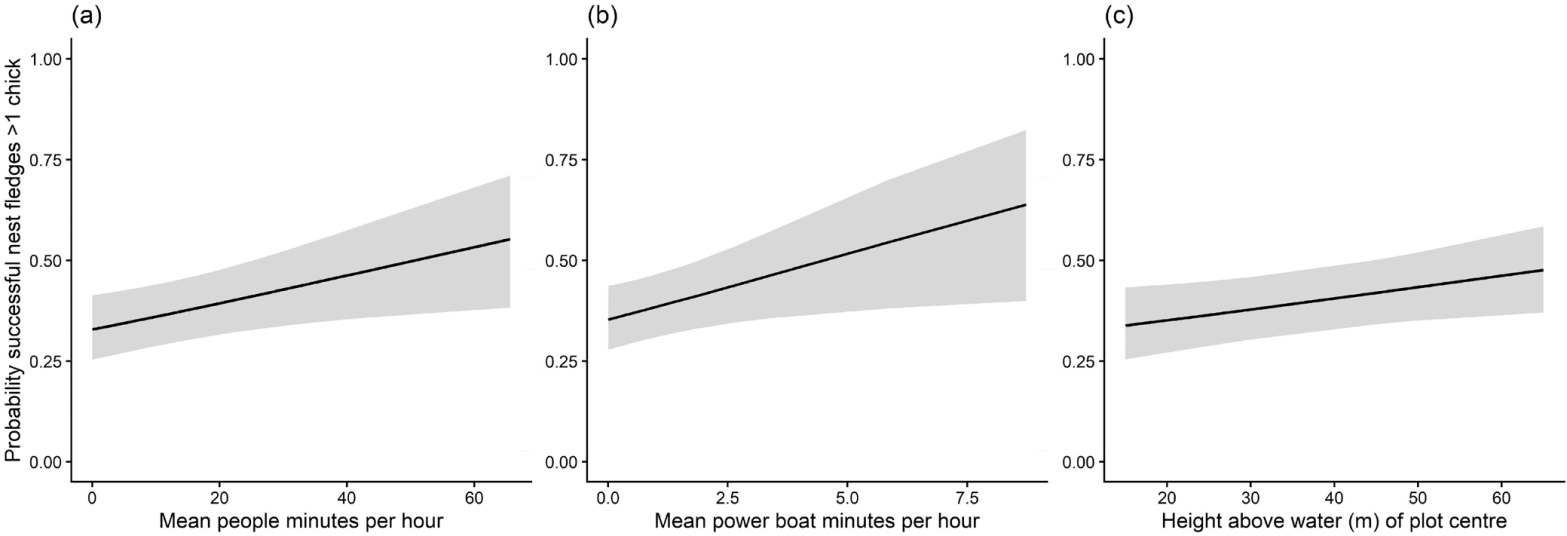
Marginal effects plots from model 60 in Table 4c showing predicted marginal effects of (a) mean people minutes per hour, (b) mean power boat minutes per hour, and (c) height above water (m) of plot centre on the probability a successful nest fledges > 1 chick.

Model‐averaged parameter estimates with 95% confidence intervals are presented in Figure 4. Effects for which their 95% confidence intervals did not overlap zero included: (i) a positive effect of people on nest success (additional analysis only) and on the number of chicks per successful nest (both analyses); (ii) a positive relationship between chicks per successful nest and power boat minutes per hour core analysis, and boat pressure scale (additional analysis); and (iii) a positive effect of plot height on chicks per successful nest (both analyses). Marginal effects from model 60 (Table 4c) are plotted in Figure 5. For brevity, only this model is presented, as it contains the same explanatory variables as model 45 from the same analysis and model 2 in Table 4d, both of which showed effects in the same positive direction. No effects from daily survival probability models are shown, as null models were also included within the confidence sets (Δ_AICc_ ≤ 2).

### 3.3 Annual Productivity and Population Sustainability

**FIGURE 6:**
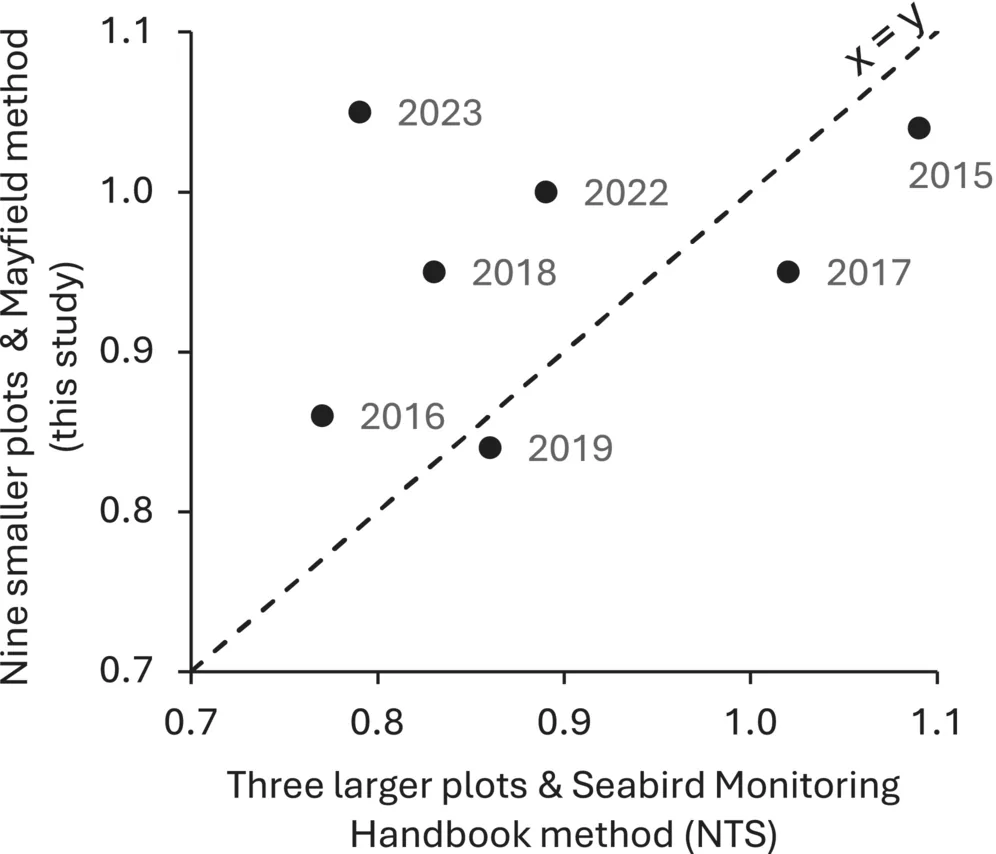
Comparison of annual estimated productivity (chicks per pair) using two different methods and a different set of plots at St Abb's Head.

There was no apparent systematic bias between the two methods of estimating seasonal productivity. In 3 years, the NTS values were higher and in 4 years, values from this study were higher (Figure 6). Differences were typically within 0.02 to 0.11 chicks per pair, except in 2023 when the NTS value (0.79) was substantially lower than that from this study (1.05).

**FIGURE 7:**
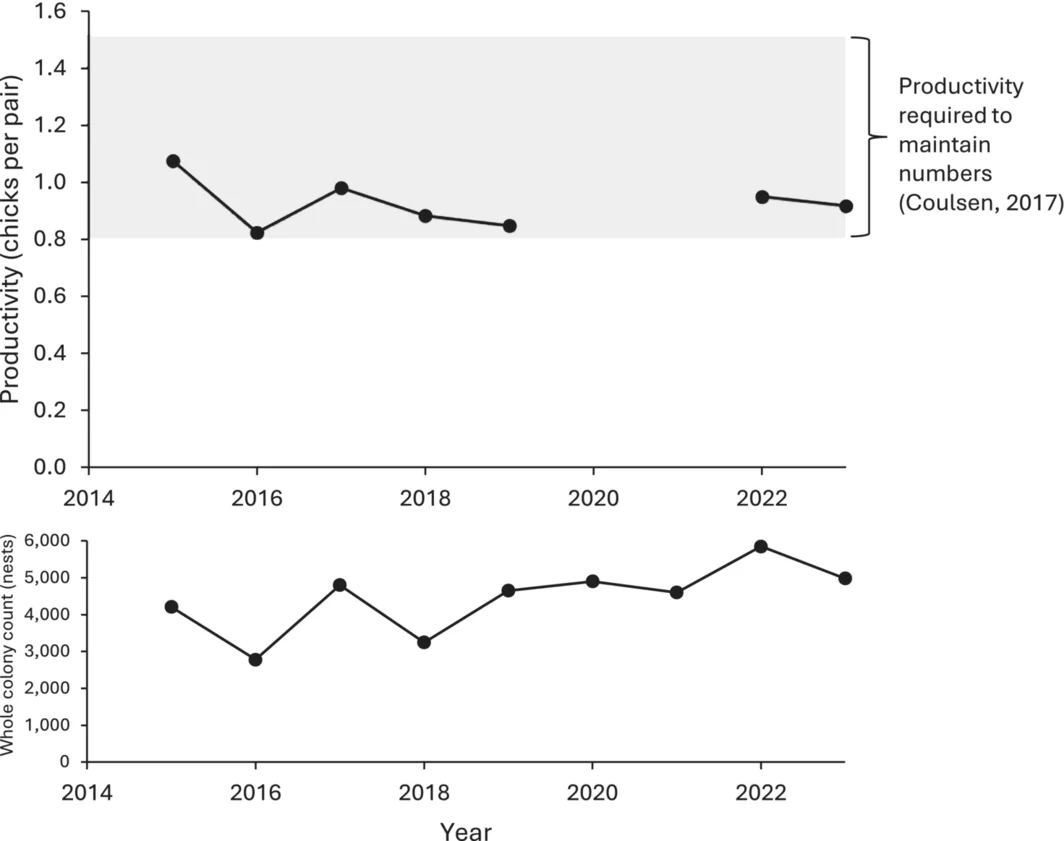
Demographic assessment of St Abb's Head NNR kittiwake population during the study period: (a) weighted‐average annual productivity estimates and (b) whole colony counts of Apparently Occupied Nests (AONs). Nest monitoring could not be conducted in 2020 and 2021 in this study, so weighted average productivity estimates are not shown for these years.

Given this lack of systematic bias, the weighted average productivity values from across both methods were plotted against the range estimated for population sustainability by Coulson (2017) (Figure 7a). All values were within that band but tended towards its lower limit. The generally flat trend within this band was consistent with the relatively stable breeding population size observed over the period (Figure 7b).

## 4 Discussion

Based on 7 years of nest monitoring data from over 1400 nests spanning wide spatiotemporal variation potential disturbance by people and boats, we found no consistent, measurable negative effects on kittiwake nest success or on numbers of fledgling per successful nest at the mainland breeding colony at St Abb's Head National Nature Reserve. Productivity, measured using our nine nest plots combined with an independent assessment at a smaller number of larger plots (combined *n* > 2800 nests), was relatively consistent between 2015 and 2023, typically between 0.8 and 1 chick per nesting pair, at the lower end of the estimate of Coulson (2017) to maintain the breeding numbers. The kittiwake population at St Abb's Head has declined from a peak of just under 20,000 occupied nests in 1989 to a relatively stable colony of about one fifth that size in the last decade, broadly reflecting national trends which are thought to have been driven by factors such as reduction in food availability and climate change impacts (Harris et al. 2024). Our results suggest that while the population does not appear to have been negatively affected by increasing people and boat traffic, it may be demographically vulnerable to future perturbations in breeding success or survival.

### 4.1 No Apparent Negative Impact of People or Boats

The absence of an apparent, measurable negative impact of people on nest success was a surprising finding. A single‐year study at St Abb's Head conducted in 2002 reported a strong negative relationship between human presence and nest success, with an 8.5% increase in people minutes associated with a 22% increase in failure rate (Beale and Monaghan 2004). That study used a different approach to quantifying people pressure, selecting the two most visible ‘viewpoints’ for each nest and calculating mean people minutes per hour from the two nearest viewpoints. In contrast, in the present study we did not select specific viewpoints but rather assessed all clifftop areas where people would be visible to birds at the centre of the plot, whether on an apparent viewpoint or not. In addition, Beale and Monaghan (2004) experimentally increased human presence by directing volunteers to visit both typically inaccessible areas and sites frequently visited by people, which on average increased the daily number of people present at the viewpoints by 11% (range 0%–100%). In contrast, in the present study we only counted people who were visiting the site passively, with no knowledge of our study. There are several possible reasons why our findings may differ. Disturbance effects may vary among years due to unmeasured environmental or ecological factors, such that impacts occur or are detectable in some years but not others. By analysing data across seven breeding seasons, such short‐term effects may not have been apparent in our study. If so, this suggests that, at least over longer timescales, kittiwake nest success is relatively resilient to human presence.

Another potential explanation for the absence of an apparent impact in our study could relate to changes in both colony size and visitor pressure over time. At the time of Beale and Monaghan's (2004) study in 2002, the kittiwake colony size (8890 apparently occupied nests) was approximately double that surveyed during our study period. In contrast, annual visitor numbers were lower in 2002, with the automated people counter recording 44,000 visitors per year (mean c. 42,000 for 1999–2005), compared with c. 57,000–97,000 visitors per year during our study period (mean c. 72,000). The methodological and contextual differences between that study and our study may have influenced apparent kittiwake responses to people presence in several ways. For example, if birds monitored during the Beale and Monaghan (2004) study were less frequently exposed to people, the experimental addition of visitors to previously inaccessible areas may have elicited stronger behavioural or physiological responses. In the intervening period, visitor numbers have almost doubled, and habituation to human presence may have occurred as has been seen in some other seabird species at their nesting colonies (Ellenberg et al. 2009). Kittiwakes, like many seabirds, are relatively long‐lived, with a typical life expectancy in Great Britain of around 12 years and a maximum recorded longevity exceeding 28 years (BTO 2025). Given that individuals typically first breed at around 4 years of age (BTO 2025), a bird may breed at St Abb's Head 8 times on average, with younger birds also likely visiting the colony prior to breeding. Such repeated exposure over extended periods may facilitate habituation to people and boat activity. Finally, given that the colony has declined by approximately half since 2002, it is possible that fewer nests now occur closer to areas where people are most visible or near cliff tops, particularly if such locations are less favourable during nest site selection.

In gull species other than kittiwakes, several studies have documented habituation to both people and boat disturbance. For example, Burger and Gochfeld (1983) reported that herring gulls and great‐black backed gulls *L. marinus* nesting in frequently human‐disturbed areas sat more tenaciously on their nests, responded more slowly, and returned to nests more quickly following an intruder's retreat, compared to birds nesting in less disturbed areas. Similarly Chatwin et al. (2013) studied the effects of boat disturbance on seabirds on Vancouver Island, including glaucous‐winged gulls *Larus glaucescens* , and found evidence of habituation in both nesting and roosting birds, with the probability of ‘agitation’ at a given boat distance decreasing as overall boat traffic increased.

Various evidence suggests that kittiwakes can tolerate close human presence at nesting colonies. Brewer et al. (2008) found no significant differences in stress‐induced levels of corticosterone levels between disturbed kittiwake chicks (nests adjacent to those visited for chick handling) and undisturbed kittiwake chicks, despite investigators abseiling to within very close proximity to nests. In Newcastle‐upon‐Tyne, UK, a kittiwake colony with more than 1400 apparently occupied nests (AONs) on human‐occupied buildings showed a nest success ranging between 61% and 75% in 2016–2019 (Turner 2019), generally higher than that recorded at St Abb's Head over the same period (Table 3). Similarly, at Dunbar (c. 26 km from St Abb's Head), a colony exceeding 1000 AONs in the early 2000s nests along an old castle wall and rock face at the entrance to an active harbour with regular people and boat traffic (Coleman et al. 2011). There, productivity fluctuated between 1.2 and 1.8 chicks per pair in most years over a 15‐year period (1990–2007). Kittiwakes also breed in highly urbanised settings, including an established colony in Lowestoft, UK, where birds nest in the town centre and dock areas (Swindells 2019).

Somewhat unexpectedly, we detected a positive relationship between both people and power boat presence and the probability that successful nests fledged more than a single chick. Acclimatisation or habituation to human presence in some seabirds may interact with predator behaviour, producing complex and sometimes counterintuitive outcomes. For example, Sandvik and Barrett (2001) examined seabird breeding performance in Norway across plots with high and low human disturbance. High‐disturbance plots received regular visits by researchers to measure chick development. In the first study year, high‐disturbance plots had reduced nest attendance and increased failure rates. However, in the second year, failure rates were substantially lower than in low‐disturbance plots. This pattern was hypothesised to reflect a shift in herring gull predation away from frequently disturbed areas and towards less disturbed colonies. Such dynamics align with one form of the ‘human shield hypothesis’, whereby human presence alters the behaviour of a ‘dominant actor’ (e.g., predator), indirectly influencing the survival or reproduction of a ‘subordinate actor’ (e.g., prey) (reviewed in Gaynor et al. 2025).

We also detected a weak positive relationship between height of plot centre and the probability that successful nests fledged more than one chick (almost always two chicks). Plot centre heights ranged from 15 to 65 m above mean sea level, representing a fourfold difference. Anecdotally, higher plots tended to be on larger faces of cliffs that rose more steeply from the water. The mechanisms underlying this pattern are unclear. No effect of distance to the cliff top was detected, suggesting that this relationship may not be directly related to proximity to humans and the human shield hypothesis described above. It has been shown that nest position in a kittiwake colony can influence adult survival and breeding success (Aebischer and Coulson 1990). However, a study in Canada reported higher attack rates by gulls on nests located in upper cliff sections compared to lower sections, an effect associated with proximity to the cliff edge rather than height above water (Massaro et al. 2001). It is therefore possible that higher plots at St Abb's Head happen to represent intrinsically favourable nest sites that are preferentially occupied by higher‐fitness individuals, although this hypothesis would require targeted testing. Additionally, higher nests may be safer from soaking by spray during summer storms, although this was not tested.

### 4.2 Sustainability of the Colony and Suitability for Compensation

Breeding success at St Abb's Head during our study (range 0.8–1.1 chicks per pair) was similar to the UK average productivity over the same period (0.6–1.1 chicks per pair; BTO et al. 2023). Both productivity and population size (c. 3000–6000) showed a relatively stable, if not slightly increasing, trend. However, population size remains low compared with the peak colony count of almost 20,000 recorded in 1989, and productivity lies towards the lower end of the range predicted to sustain populations in the long term (Coulson 2017). As a result, the population may be demographically vulnerable, such that any future declines in either juvenile or adult survival, or in productivity, could tip the population into decline.

Recently, the Berwick Bank offshore windfarm was consented (i.e., formally approved for construction), with a generating capacity of up to 4.1 GW of electricity, making it one of the largest offshore windfarms in the world (Scottish Government 2025c; SSE Renewables, n.d.). However, this consent is subject to the developer producing a suitable ‘Seabird Compensation Plan’ to detail how predicted adverse impacts on seabirds will be compensated for (Scottish Government 2025c). The closest edge of the turbine array would lie approximately 35 km from St Abb's Head. Under the Habitats Regulations Assessment, kittiwakes nesting at St Abb's Head were screened in for ‘Likely Significant Effects’ during the construction, operation, maintenance and the decommissioning phases (Scottish Government, n.d.). Potential impacts were identified from disturbance and displacement, accidental pollution, changes in prey availability and barriers to movement. Multiple scoping approaches were applied and, within the Environmental Impact Assessment, cumulative displacement‐related mortality was estimated to increase 0.55%–1.63% across all seasons. These effects were assessed as ranging from ‘minor’ to ‘moderate’ adverse significance, the latter being considered ‘significant’ in EIA terms. A population viability analysis using a seabird specific model (Searle et al. 2022) further explored potential consequences under a range of scenarios relating to windfarm development at the Berwick Bank within the Forth and Tay region and across the wider North Sea. Under these scenarios, the modelling predicted declines in the breeding kittiwake population of the St Abb's Head to Fast Castle Special Protection Area (which includes St Abb's Head). Relative to counterfactual scenarios with no windfarm impacts, the colony was predicted to be between one‐third and one‐half of its no‐impact‐size by 2062, and between one‐quarter and one‐third by 2077, based on median model predictions.

Given that a recent consultation has proposed a ‘reduction in disturbance to colony’ as a form of wider compensation measure for mortality associated with offshore windfarms (Scottish Government 2025b), caution is required when assuming that reductions in disturbance from people and boats would necessarily deliver meaningful demographic benefits for kittiwakes. Our findings indicate considerable uncertainty as to whether, under current levels of people and boat activity at the site (and the potential that kittiwakes have become habituated to those numbers), that any disturbance reduction measures would produce any demographically meaningful impact for this species. Caution is advised, however, that this would apply to every colony, as disturbance impacts may be mediated by factors such as how habituated birds are to people and the proximity of people and boats to their nests. In addition, other species at St Abbs Head might show different responses. Thus, our results do not mean that where there are acute issues of disturbance such as very close approaches to nesting birds that these shouldn't be addressed. For example, we have more recent evidence of negative behavioural and displacement impacts of powered and unpowered boat approaches on guillemots, both at nest sites and inshore loafing areas on the water (unpublished data).

Other compensation or mitigation measures that have been proposed or implemented for kittiwakes in response to predicted impacts of offshore windfarms include rat *Rattus* spp. eradication at island nesting colonies, sandeel fishery cessation or management measures, construction of artificial nesting sites, and employment of wardens at colonies (Rhoades et al. 2025; SSE Renewables 2023). It is important that the demographic impacts of such measures are robustly monitored to provide evidence to inform future compensation or mitigation approaches in offshore windfarm plans. Responses to the Scottish Government's public consultation on the Strategic Compensation Policy for Offshore Wind supported this stance, calling for ‘robust, peer‐reviewed evidence and independent scientific review… to support claims of ecological benefit’ (Scottish Government 2025d). Recent statutory guidance also requires that ‘appropriate evidence‐based justifications’ are used for proposing compensation measures (Scottish Government 2026c).

## 5 Conclusions

With the current and future large‐scale expansion of offshore windfarms in the North Sea, there is increasing interest in strategic compensation for losses to the UK's internationally important breeding seabird populations. Disturbance is identified as a key threat to safe breeding and foraging habitats in the Scottish Seabird Conservation Action Plan, with minimisation of disturbance at colonies through guidance and seasonal staffing listed as a core management measure (Scottish Government 2025e). In parallel, the Scottish Government now has policy and regulations in place for evidence‐based, strategic‐level compensation for offshore windfarms (Scottish Government 2026c, 2026b), which can include measures aimed at reducing disturbance at seabird colonies. Our longitudinal study at one of the UK's most important mainland seabird colonies, close to the planned site of what will be one of the world's largest offshore windfarms, detected no negative relationship between exposure to people or boats and kittiwake nest success and identified a weak positive relationship with the probability that successful nests fledged more than a single chick. This contrasts with earlier work and may indicate habituation to human presence or a ‘human shield’ interaction between people and nest predators. While disturbance can pose acute risks and responses are likely to differ among species, our findings suggest that disturbance‐focussed mitigation or compensation measures are unlikely to deliver demographically meaningful benefits for kittiwakes at St Abb's Head. As such, alternative compensation measures aimed at enhancing breeding success, recruitment or survival would be required.

## Author Contributions

**Patrick J. C. White:** conceptualization (lead), data curation (lead), formal analysis (lead), investigation (lead), methodology (lead), project administration (lead), supervision (lead), visualization (lead), writing – original draft (lead), writing – review and editing (lead). **Hannah Humphreys:** data curation (lead), investigation (equal), writing – review and editing (supporting). **Jill M. Grozier:** data curation (equal), investigation (equal), writing – review and editing (supporting). **Jenna R. Lane:** data curation (equal), investigation (equal), visualization (equal), writing – review and editing (supporting). **Alexander M. Willey:** data curation (equal), investigation (equal), writing – review and editing (supporting). **Annalise Bokenkamp:** data curation (equal), investigation (equal), writing – review and editing (supporting). **Nils H. Schoefer:** data curation (equal), investigation (equal), writing – review and editing (supporting). **Sophie J. Wilson:** data curation (equal), investigation (equal), writing – review and editing (supporting). **Ross J. Rutherford:** data curation (equal), investigation (equal), writing – review and editing (supporting). **Liza Cole:** conceptualization (equal), project administration (equal), resources (supporting), supervision (supporting), writing – review and editing (supporting). **Ciaran Hatsell:** project administration (equal), resources (equal), supervision (supporting), writing – review and editing (supporting). **Rachel Bonnici:** project administration (equal), resources (equal), supervision (supporting), writing – review and editing (supporting). **Ellie Owen:** project administration (equal), resources (equal), supervision (supporting), writing – review and editing (supporting). **Michelle Frost:** investigation (equal), supervision (equal), writing – review and editing (equal). **Sue Lewis:** investigation (equal), supervision (equal), writing – review and editing (equal). **Karen Diele:** conceptualization (lead), funding acquisition (lead), investigation (lead), methodology (lead), project administration (lead), supervision (lead), writing – review and editing (lead).

## Funding

This work was supported by The AEB Charitable Trust.

## Conflicts of Interest

The authors declare no conflicts of interest.

## Figures and Tables

**TABLE A1:**
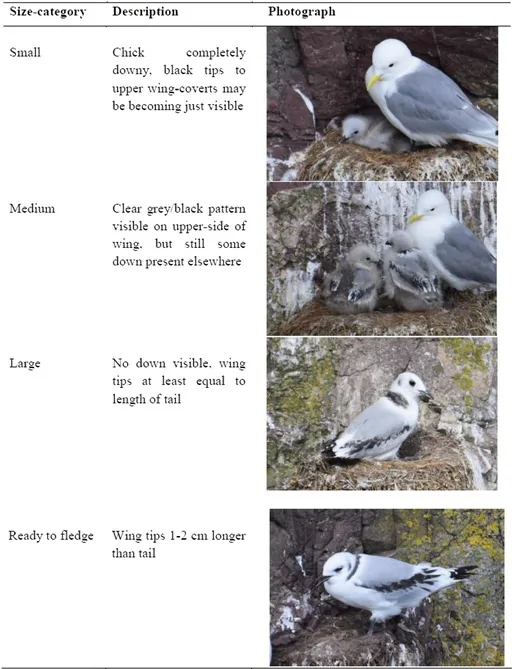
Kittiwake chick age categories adapted from the JNCC Seabird Monitoring Handbook of Britain and Ireland (Walsh et al. 1995). Photos by JL.

## Data Availability

The data that support the findings of this study are openly available in Zenodo at https://doi.org/10.5281/zenodo.20325904, v.1.0.1.

## Appendix A

### Categorisation of Size‐Category of Kittiwake Chicks

See Table A1.

## Appendix B

### Calculation of Scale People and Boat Pressure Variables

For the ‘additional analysis’ stratum, ‘scaled people pressure’ (SPP) and ‘scaled boat pressure’ (SBP) variables for each plot in each year were calculated as follows:

(A1)
$$\mathrm{SPP}=\left({\text{people}}_{\text{plot}}-{\text{people}}_{\min}\right)/\left({\text{people}}_{\max}-{\text{people}}_{\min}\right)$$

(A2)
$$\mathrm{SBP}=\left({\text{boats}}_{\text{plot}}-{\text{boats}}_{\min}\right)/\left({\text{boats}}_{\max}-{\text{boats}}_{\min}\right)$$

where people_plot_ is the value of the people variable for that plot in a given year, people_min_ is the minimum value of the people variable across all plots in a given year, and people_max_ is the maximum value of the people variable across all plots in a given year, with the same notation for boats. Essentially, the plot with the highest value of people or boats (regardless of how calculated) in a given year was assigned a value of 1, and the plot with the lowest was assigned a value of 0, with intermediate values scaled between these.
